# Legislative Social Reforms

**Published:** 1888-05-26

**Authors:** 


### The Hospital

A Weekly Institutional Journal of

Science, Medicine, Nursing, and Philanthropy.

*For Hospitals, Asylums, Medical Practitioners, Students, Nurses, Families, Parishes, Congregations, and the Charitable Public.*

Vol. IV. No. 87.] [May 26, 1888.

### Legislative Social Reforms.

A significant meeting was held at Grosvenor House, under the presidency of the Earl of Meath, on Thursday week. The meeting consisted exclusively of members of the two Houses of Parliament, and was convened for the purpose of making a beginning to turn into concrete facts some of the ideas which intelligent social reformers hold as convictions, and grieve over as, at present, mere powerless abstractions. It is not surprising that earnest men, like Lord Meath and the other members of the Legislature who think with him, should despair of the efficacy of magazine and newspaper articles, and long to put their hands to actual effort, in order to realise at once—what they believe to be contained as seeds in their abstract principles—the well being and happiness of their poorer and less fortunate fellow men. Instruction is a slow process of improvement; and when there are many instructors, each with a different set of infallible rules, and each crying loudly that his are the truest, the best, and indeed the only rules worthy of serious consideration, the pupils are apt to conclude that as many of the rules are mutually destructive, so they must be all alike unnecessary and useless. That is not so. But the result of the operations of too many cooks is not infrequently disastrous.

Carlyle, wearied with the babble of tongues and of the futile results of manifold discussion, longed for a wise and beneficent despotism. The people need guidance, they need government; where is such guidance to be found? Catch your able man, said Carlyle, and when you have caught him, give him a free hand; give him authority, give him resources; let him realise in bodily form the visions of his seeing and creative mind. But that cannot be, at any rate here in England and now. Hardly any age has the skilled vision to see its own competent man; still less has it the self-denying nobility of disposition to raise him aloft and give him the sceptre of power. This generation can single out the great and heroic men of three generations ago; and probably our grandchildren may have succeeded in rightly placing the men of this generation; but that will be too late for immediate practical advantage. Those of us who believe in the efficacy of a wise and strong government, have profound sympathy with Carlyle's desire for a wise and effective despotism.

Failing the autocrat—what next? One true ruler would be worth a thousand mere talkers; but ten true rulers would be greatly more valuable than one. Is that the solution of the difficulty? Are the men who have joined with the Earl of Meath of such an excellently wise and kingly character that they will unitedly take in hand and guide the rudderless ships which founder on all hands for want of such guidance? That remains to be seen. The one prime requisite for service it may at once be admitted they possess—an honest and intense desire to help their fellow men.

What do the Earl of Meath and his sympathisers propose to do? Not to form a new debating society. Academical discussions on social questions are played out. Nobody cares one straw for any other Parliament than that at Westminster; and, similarly, no interest is excited in the discussions of any reforming association which does not intend to put its hand to actual work. The new social reformers have done something at their first meeting. They have formed a Parliamentary organisation, independent of party politics. Too much approval can hardly be expressed of this first step. The organisation has taken the form of a committee, and consists exclusively of members of one or the other House of Legislature. That, too, is well; at any rate, at the outset. It may be necessary to include special persons from among the general public for special objects at a later stage.

Having got their organisation, what is the one guiding principle and practical aim of its existence? To promote social reforms by means of legislation. It must be emphasised that the reforms to be promoted are not political nor constitutional, but social. That is to say, Parliament is to be called upon by those who have authority and influence behind them, and who have a following in the country, to do what it seldom, with all its willingness, has had the energy to undertake. It is said that there are only two subjects in the world outside the average man's own personal affairs which have any living and quickening interest for him. Those two subjects are Religion and Politics. Of these subjects we admit at once the great, the paramount importance. But in an age like the present, of crowded populations, keen competitions, hand-to-hand struggles for existence, innumerable failures in the struggle, fallings out of the ranks, despairing and futile efforts to recover lost ground, utter and heart-rending defeats—in such an age he would be less than human and unworthy of the name of Christian who should have no desire to help the struggling up again, no longing to put more hope into the conflict, no determination to make victory at least possible to every man who has the courage and the energy to deserve it.

"The whole question of social reform is now in the air," said Mr. Samuel Smith, himself one of the members of the new organisation. "The society was not wanted to stimulate the country so much as to guide and direct it wisely." There were "desperate evils to deal with in the social conditions of the people. The shocking revelations in connexion with the sweating dens made us feel that we were in the midst of probably as deep a stratum of misery as ever existed in the world's history." There were, he thought, two directions which social reform ought to take—temperance reform and the protection of the young. In the circular issued explanatory of the objects of the meeting, and which was signed by ten peers and eight members of the House of Commons, it was stated that "the consideration of social and philanthropic reform had not received in the past that share of interest within the walls of Parliament which should be accorded to it. . . . . Pauperism, workhouse reform, starvation wages, over-population, foreign immigration, colonization, emigration, pollution of air and water, prevention of infection, the drink and opium questions, overcrowding of dwellings, unsanitary buildings, the social evil question, early closing; industrial, technical, commercial and physical education; public parks and playgrounds; factory, mining, and workshop legislation and inspection; small holdings, allotments, national insurances"—all these and other such topics of urgent practical moment to millions of the people can hardly get a hearing in authoritative quarters.

That such is the fact no reader of current news for a moment doubts; that such neglect of affairs which concern the happiness and well-being of millions of the more helpless portions of the community, is calamitous, no one with the least degree of intelligence will deny: that so many members of both Houses of Parliament, who have already gained the confidence of the public on social questions in an unusual degree, should combine and resolve to throw themselves into the breach, and compel attention and consideration is a circumstance of great significance, and full of promise that better days are at hand. The new association is composed of men of means, of leisure, of education, and of an intelligent sense of duty. Its members have the ear of the country; they have each a following more or less strong behind them: individually they can demand a hearing in Parliament, unitedly they can compel it. What will be the practical outcome of their labours within the next five years? If they have accomplished nothing, or next to nothing, within that period, we may despair of collective efforts at social amelioration. It may then be wise to shut our eyes fast and wait stolidly until natural forces and the "dismal science" work out social salvation or social ruin for the masses of our fellow countrymen.

